# Central Administration of Lipopolysaccharide Induces Depressive-like Behavior *in Vivo *and Activates Brain Indoleamine 2,3 Dioxygenase In Murine Organotypic Hippocampal Slice Cultures

**DOI:** 10.1186/1742-2094-7-43

**Published:** 2010-08-02

**Authors:** Xin Fu, Samantha M Zunich, Jason C O'Connor, Annemieke Kavelaars, Robert Dantzer, Keith W Kelley

**Affiliations:** 1 Integrative Immunology and Behavior Program, Department of Animal Sciences, College of ACES, University of Illinois at Urbana-Champaign, Urbana, Illinois 61801, USA; 2 Department of Pathology, College of Medicine, University of Illinois at Urbana-Champaign, Urbana, Illinois 61801-3873, USA; 3 Laboratory of Neuroimmunology and Developmental Origins of Disease (NIDOD), University Medical Center Utrecht, 3584 EA Utrecht, The Netherlands

## Abstract

**Background:**

Transient stimulation of the innate immune system by an intraperitoneal injection of lipopolysaccharide (LPS) activates peripheral and central expression of the tryptophan degrading enzyme indoleamine 2,3 dioxygenase (IDO) which mediates depressive-like behavior. It is unknown whether direct activation of the brain with LPS is sufficient to activate IDO and induce depressive-like behavior.

**Methods:**

Sickness and depressive-like behavior in C57BL/6J mice were assessed by social exploration and the forced swim test, respectively. Expression of cytokines and IDO mRNA was measured by real-time RT-PCR and cytokine protein was measured by enzyme-linked immunosorbent assays (ELISAs). Enzymatic activity of IDO was estimated as the amount of kynurenine produced from tryptophan as determined by high pressure liquid chromatography (HPLC) with electrochemical detection.

**Results:**

Intracerebroventricular (*i.c.v*.) administration of LPS (100 ng) increased steady-state transcripts of TNFα, IL-6 and the inducible isoform of nitric oxide synthase (iNOS) in the hippocampus in the absence of any change in IFNγ mRNA. LPS also increased IDO expression and induced depressive-like behavior, as measured by increased duration of immobility in the forced swim test. The regulation of IDO expression was investigated using *in situ *organotypic hippocampal slice cultures (OHSCs) derived from brains of newborn C57BL/6J mice. In accordance with the *in vivo *data, addition of LPS (10 ng/ml) to the medium of OHSCs induced steady-state expression of mRNA transcripts for IDO that peaked at 6 h and translated into increased IDO enzymatic activity within 8 h post-LPS. This activation of IDO by direct application of LPS was preceded by synthesis and secretion of TNFα and IL-6 protein and activation of iNOS while IFNγ expression was undetectable.

**Conclusion:**

These data establish that activation of the innate immune system in the brain is sufficient to activate IDO and induce depressive-like behavior in the absence of detectable IFNγ. Targeting IDO itself may provide a novel therapy for inflammation-associated depression.

## Background

Clinical and animal studies implicate systemic inflammation in the pathogenesis of major depressive disorders by activating immune-to-brain communication pathways [1-5]. Proinflammatory cytokines induce symptoms of sickness that correspond to a temporary reorganization of the organism's priorities to cope with infections. If the neuroimmune inflammatory response does not resolve, sickness behavior can culminate in depressive-like behavior [2,6]. Inflammation-induced depression is associated with activation of the tryptophan degrading enzyme indoleamine 2,3 dioxygenase (IDO) in both the brain and periphery. IDO is a ubiquitous enzyme that degrades tryptophan into kynurenine, which can decrease the bioavailability of tryptophan for the synthesis of serotonin [7-11]. The kynurenine/tryptophan ratio increases in the plasma and brain of patients and animals following both acute and chronic stimulation of the immune system [12-17]. Kynurenine can be further degraded into a number of neuroactive metabolites that act as agonists or antagonists of the NMDA receptor that can ultimately impact on glutamatergic neurotransmission [18-20].

Sickness is an adaptive response to infectious pathogens. Although sickness and depression can share symptoms, the similarities are only partial. Sickness rather than depression is fully reversible once the pathogen has been eliminated [2]. In both mice injected with lipopolysaccharide (LPS) or infected with *Bacille Calmette-Guerin*, depressive-like behavior remains after sickness behavior had abated [2]. Systemic administration of LPS is a well-established model to study behavioral and physiological responses following peripheral activation of the immune system. Sickness behavior peaks in the first 2 to 6 h following intraperitonal injection of LPS and gradually returns to normal [2,14,21]. Twenty four hours after treatment, when sickness behavior has diminished, depressive-like behavior still remains, as measured by increased immobility in both the forced swim test (FST) and tail suspension test and decreased preference for a sweetened solution [2,14,21]. These findings indicate that the LPS model of peripheral acute immune activation can dissociate sickness from depression.

Activation of IDO is a key event in the switch from sickness to depression. Blockade of cytokine production and IDO activation by administration of the anti-inflammatory tetracycline derivative minocycline prevents both LPS-induced sickness behavior and depressive-like behavior [14,22]. Administration of the IDO competitive inhibitor 1-methyl tryptophan abrogates development of LPS-induced depressive-like behavior without altering LPS-induced expression of proinflammatory cytokines and the development of sickness behavior [14].

In all the experiments carried out to date with systemic administration of LPS, IDO is activated both peripherally and in the brain. Peripherally activated IDO produces large quantities of kynurenine that can enter the brain via the same transporter as that for tryptophan [23,24]. This is important because peripherally administered kynurenine can induce depressive-like behavior in naive mice [14]. Although IDO is predominantly expressed in peripheral organs such as the lungs, it is also present in the brain where it can be activated by immune and non-immune stimuli [2]. However, the exact brain structures *in situ *where IDO is activated are less well known. Classic neurocircuitry of depression often refers to cortical-striatal-limbic networks of neural circuits that involve the sub-genual anterior cingulate cortex [25]. However, it is less appreciated that the hippocampus is another brain area that plays a role in the pathophysiology of major depressive disorders. It has been extensively studied in patients with mood disorders [26,27] and is an important structure in the neurogenesis theory of depression [26,28]. In mice, the hippocampus is also related to depressive-like behavior [21,29]. Indeed, IDO is activated in the mouse [22,29,30] and rat [31] hippocampus following systemic administration of LPS. It is not yet known whether LPS administered directly in the cerebral ventricles is able to activate IDO in the hippocampus concomitantly with its ability to induce depressive-like behavior. Therefore, one of the questions we sought to answer is whether activation of IDO in the brain is sufficient to induce depressive-like behavior.

In order to study the mechanisms involved in the activation of IDO by central administration of LPS, we used organotypic hippocampal slice cultures (OHSCs) that have the advantage of preserving the basal cellular and connective organization of this brain area and several fundamental *in vivo*-like characteristics such as glial-neuronal interactions [32,33]. This preparation has previously been used for studying the detrimental effects of proinflammatory cytokines on long-term potentiation in the rat system [34,35]. We show for the first time that centrally administered LPS is sufficient to activate IDO in the hippocampus and induce depressive-like behavior. Using organotypic hippocampal slice cultures we further demonstrate that this brain area responds to LPS by increased expression of IDO and cytokines, followed by activation of IDO. Finally, both the *in vivo *and *in situ *data support the idea that IDO can be regulated in an IFNγ-independent manner [36,37]. These new findings point to the role of brain IDO in inflammation-induced depression.

## Methods

### Reagents

LPS was from *Escherichia coli *0127:B8 (cat# L-3137). Heat-inactivated horse serum (cat# SH30074.03), Hank's balanced salt solution (HBSS, cat# SH30030.03) and MEM (cat# SH30024.02) were all from Hyclone. Gey's balanced salt solution (GBSS, cat# G9779) was from Sigma. D-glucose (cat# 15023-021) was from GibcoBRL and propidium iodide (PI, cat# P3566) was from Molecular Probes Inc. (Eugene, OR). Kits for enzyme-linked immunosorbent assay (ELISA) were from R&D Systems (Wiesbaden, DE). TRIzol reagent was from Invitrogen Life Technologies (Carlsbad, CA). Reagents for RT-PCR were all from Applied Biosystems as follows: Ambion (cat# 1710) reverse transcriptase kit, Ambion's DNA-free™ DNase treatment and removal reagents (cat# AM1906), RT-PCR primers for TNFα (cat# Mm00443258_m1), IL-6 (cat# Mm00446190_m1), IFNγ (cat# Mm00801778_m1), IDO (cat# Mm00492586_m1), the inducible isoform of nitric oxide synthase (iNOS) (cat# Mm00440485_m1) and glyceraldehyde-3-phosphate dehydrogenase (GAPDH; cat# Mm999999_g1). The protease inhibitor cocktail (cat# P2714) was from Sigma.

### Mice

All animal care and use procedures were conducted in accordance with the Guide for the Care and Use of Laboratory Animals (National Research Council) and approved by the Institutional Animal Care and Use Committee. Experiments conducted *in vivo *were performed on 8-12-week-old male C57BL/6J mice obtained from a colony raised in our laboratory. Mice were individually housed in standard shoebox cages, with wood shavings as litter, in a temperature- (23°C) and humidity- (45-55%) controlled environment with a 12/12 h modified dark-light cycle (lights on 10:00 P.M.-10:00 A.M.). Food and water were available *ad libitum*.

### Intracerebroventricular (i.c.v.) Cannulation

A stainless steel guide cannula (23-gauge, 7 mm length) was surgically implanted unilaterally 0.5 mm above the lateral ventricle of the brain, as previously described [38]. After anesthetizing with ketamine and xylazine (1 mg and 0.1 mg/10 g body weight *i.p*., respectively), mice were secured in a Kopf stereotaxic instrument (Tujunga, CA, USA). Coordinates for placement of the guide cannula were 1 mm posterior to bregma, 1.6 mm lateral and 2.0 mm below the skull surface at the point of entry. Mice were allowed to recover a minimum of 7 days before treatment and initiation of behavioral tests. After recovery, mice were slowly injected over 1 min *i.c.v*. with sterile saline or LPS (100 ng) in a volume of 1 μl. This dose of LPS was selected on its ability to reliably induce sickness behavior [37-39]. The cannula was left in place for 1 min following injection.

### Behavioral Experiments

All behavioral experiments were performed during the first 4 h of the dark phase of the light cycle and were video taped.

#### Social exploration behavior

Social exploration was carried out before and then 4, 8, and 24 h post *i.c.v*. injection using a protected novel juvenile that was introduced into the test subject's home cage for a five-min period. Social behavior was determined as the amount of time that the experimental subject spent investigating the juvenile [38].

#### Forced swim test

The forced swim test was conducted at 24 h post *i.c.v*. injection for a six-min period. Immobility was defined as passive floating behavior or any movement necessary for the mouse to keep its head above water, as described previously [30].

### Organotypic Hippocampal Slice Cultures

Hippocampal slice cultures were prepared using the static interface culture method [39]. Briefly, 6- to 8-day-old C57BL/6J mice were euthanized by decapitation. The brains and meninges were removed, followed by separation of the hippocampus from both hemispheres. Hippocampi were dissected and transverse slices (350 μm in thickness) were prepared with a McIlwain tissue chopper (Campden Instruments Ltd, UK). Slices were placed for 1 h at 4°C into GBSS supplemented with 2 mg/ml **D**-glucose and were then transferred onto porous (0.4 μm) transparent membrane inserts (30 mm in diameter; Millipore) with five slices on each insert. Inserts were then placed into six-well culture plates. Each well contained 1.2 ml of nutrient medium composed of 25% heat-inactivated horse serum, 25% HBSS and 50% MEM, supplemented with 25 m**M D**-glucose. Neither antibiotics nor anti-mitotics were used. Plates were maintained in a humidified CO_2 _incubator (5% CO_2_, 95% atmospheric air) at 37°C. Medium was changed every 2-3 days. The MEM medium was changed so that it contained only 5% horse serum and 25 mM D-glucose on the day that LPS was added. At various times following addition of LPS, supernatants were collected and stored at -80°C for measurement of cytokines. Slices were washed twice with cold PBS and stored at -80°C for isolation of total cellular RNA and determination of IDO enzymatic activity.

### Slice staining

Brain slices were stained at several times after sectioning by adding PI to label dead cells. Slices were incubated at 37°C for 30 min in 2 μM PI. Slices were then imaged using an Axiovert 40 CFL microscope equipped with Axio CamMR using a 2.5× objective. Measurement of the intensity of PI staining was quantified using AxioVision.

### Reverse transcription and Real-time RT-PCR

Total cellular RNA from the hippocampus and cultured slices was extracted in TRIzol reagent, as previously described [16]. Total mRNA (1-2 μg) was reverse transcribed to cDNA using reverse transcriptase kits from Ambion. Samples were run in duplicate. Data were analyzed using the comparative threshold cycle method, as described elsewhere (Applied Biosystems User Bulletin no.2).

### Enzyme-linked immunosorbent assays (ELISAs)

TNFα and IL-6 were measured with validated specific ELISA assays according to the manufacturer's instructions. Briefly, 100 μl of each sample was added in duplicate to ELISA plates pre-coated with an anti-TNFα or IL-6 capture antibody. Recombinant murine TNFα and IL-6 standards ranged from 0 to 1,000 pg/ml. The lower assay limit of detection was 16 pg/ml. Absorbance was measured on an OPTImax ELISA plate reader. TNFα and IL-6 concentrations were expressed as picograms per milliliter.

### Determination of IDO activity

IDO activity was measured as previously described [40], with minor modifications. Briefly, slices were disrupted with ice-cold lysing buffer (140 mM KCl, 20 mM potassium phosphate buffer, pH 7.0) with a cocktail of protease inhibitors. After centrifugation of the homogenates (13,000 × *g*, 10 min, 4°C), supernatants were incubated for 1 h at 37°C in the following reaction mixture: 0.4 mM L-TRP, 20 mM ascorbate, 10 μM methylene blue, 100 μg catalase in 50 mM phosphate buffer, pH 6.5. The reaction was stopped by addition of 10% sulfosalicylic acid solution (SSA), followed by incubation for 30 min at 50°C to convert N-formylkynurenine to L-kynurenine (L-KYN). After centrifugation (13,000 × *g*, 10 min, 4°C) and ultrafiltration (0.2 μM filter tubes), the amount of L-KYN produced from TRP was determined by High Pressure Liquid Chromatography (HPLC) with electrochemical detection, as previously described [14]. Enzymatic activity was expressed as the product content per hour per milligram of protein.

### Statistical analysis

Data from social exploration were analyzed using a two-way (treatment × time) ANOVA with repeated measures on the time factor. Data from other experiments were analyzed using a one-way (treatment) ANOVA, followed by a Fisher's LSD *post hoc *test if the main effect was significant. All data are presented as means ± SEM. Differences were considered significant if the probability reached a level of 0.05 or smaller.

## Results

### Central LPS challenge induces an episode of sickness behavior followed by depressive-like behavior

**Figure 1:**
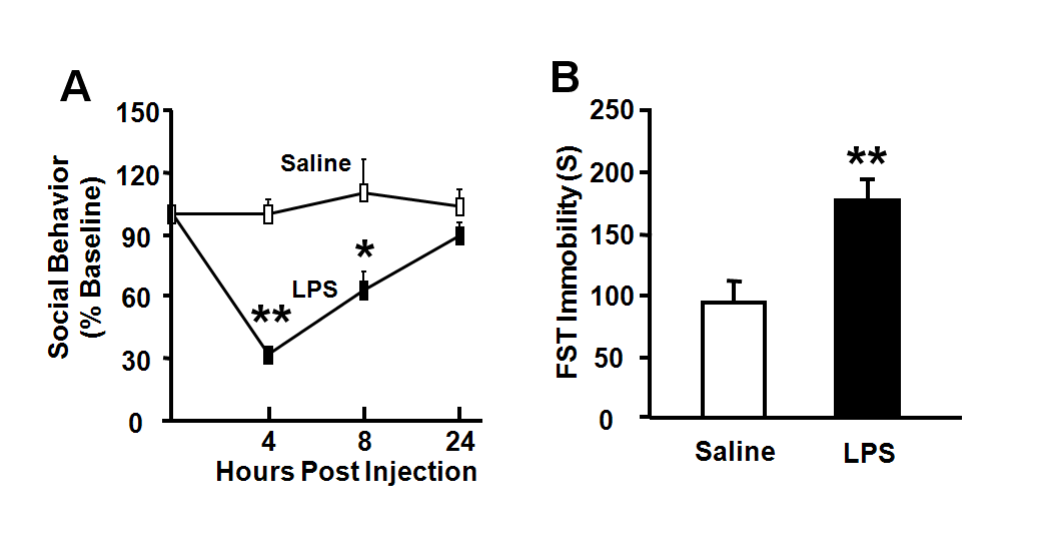
**LPS administered via the *i.c.v*. route induces both sickness and depressive-like behavior**. Mice were injected *i.c.v*. with either saline or LPS (100 ng). (A) Social behavior was measured during a 5 min period at 0, 4, 8 and 24 h following LPS injection. (B) Duration of immobility was quantified during a 6 min forced swim test that was recorded 24 h following administration of LPS. Data represent mean ± SEM (n = 4-6 mice/group). * *p *< 0.05, ** *p *< 0.01 compared to saline.

Peripheral LPS injection causes depressive-like behavior at 24 h in the absence of sickness response [14,21], so we sought to determine if similar behavioral changes could be induced by centrally administered LPS at a dose (100 ng) that we previously established to induce sickness behavior in both CD1 [41] and C57BL/6J [42,43] mice. LPS-induced sickness was measured by assessing body weight and social exploration. As expected, *i.c.v*. administration of LPS induced a significant reduction in body weight 6 h after LPS injection compared to saline-treated controls (-0.4 ± 0.3 (n = 4) vs. 0.5 ± 0.1 g (n = 5); *p *< 0.05). LPS reduced social exploration in a time-dependent manner (treatment × time: *p *< 0.01). Social exploration was reduced at 4 h (*p *< 0.01) and 8 h (*p *< 0.05), returning to baseline exploration by 24 h after *i.c.v*. injection of LPS (Fig. 1A). In contrast, LPS increased immobility in the FST at 24 h post-treatment (Fig. 1B, *p *< 0.01). Collectively, these data establish that acute activation of the central innate immune system with LPS in mice induces depressive-like behavior that does not overlap with sickness behavior.

### Central LPS challenge induces IDO, cytokine and iNOS expression in the hippocampus

**Figure 2:**
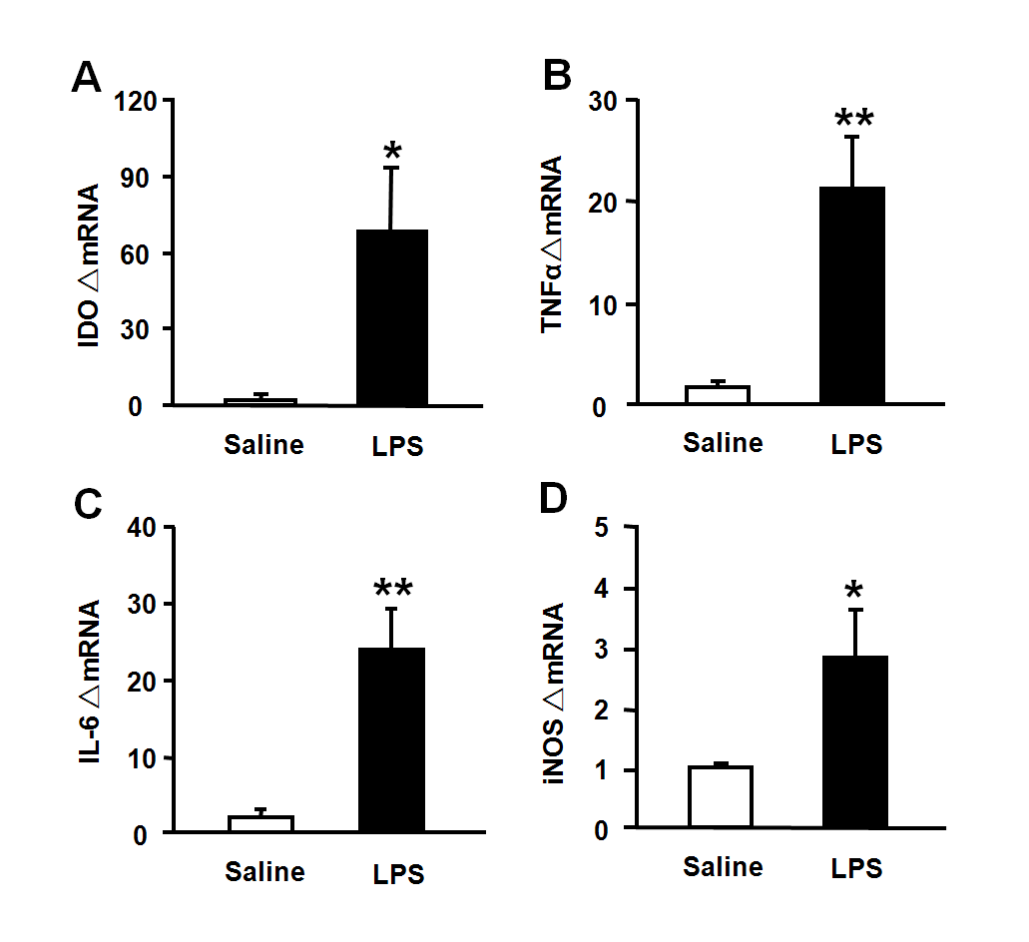
**Central administration of LPS induces (A) IDO, (B) TNFα, (C) IL-6 and (D) iNOS mRNA upregulation in the hippocampus**. Mice were injected *i.c.v*. with either saline or LPS (100 ng) and the hippocampi were collected 6 h later. Steady-state expression of mRNA transcripts was measured by real-time RT-PCR. Average Ct values for LPS-treated mice were as follows: IDO = 33.0 ± 0.4; TNFα = 23.3 ± 0.4; IL-6 = 24.2 ± 0.4; iNOS = 28.0 ± 0.3. Data represent mean ± SEM (n = 4-5 mice/group). * *p *< 0.05, ** *p *< 0.01 compared to saline.

We have established that IDO and cytokine expression in the hippocampus of mice injected *i.p*. with LPS peaks within 6 h [29]. Therefore, the hippocampus was collected at the 6 h time point to determine the effects of LPS injected *i.c.v*. on the brain. Although IDO mRNA was undetectable in saline-treated mice, its expression increased significantly following central injection of LPS (*p *< 0.05) (Fig. 2A). LPS also increased TNFα (*p *< 0.01; Fig. 2B), IL-6 (*p *< 0.01; Fig. 2C), and iNOS (*p *< 0.05; Fig. 2D) mRNA expression compared to saline-injected controls. LPS did not cause a statistically significant increase in IFNγ mRNA expression (data not shown). These results indicate that centrally administered LPS activates brain cytokine signaling and increases brain IDO expression.

### Hippocampal slices largely recover from explantation by day 7 in tissue culture

In order to investigate the mechanisms responsible for inflammation-induced depression, organotypic cultures of hippocampal slices were prepared from 6 to 8-day-old C57BL/6J mice. Preparation of murine hippocampal slices from postnatal brain requires cutting the hippocampus. We reasoned that the trauma caused by removal of afferents and efferents induced by the transversal slicing of hippocampus would cause an acute, but not chronic, inflammatory reaction. In order to assess the length of time required by OHSCs to recover, we collected both tissue slices and media at various time points to measure mRNA expression and protein levels of proinflammatory cytokines using real-time RT-PCR and ELISA, respectively. LPS (10 ng/ml; 6 h treatment) was used as a positive control to treat OHSCs on day 10.

**Figure 3:**
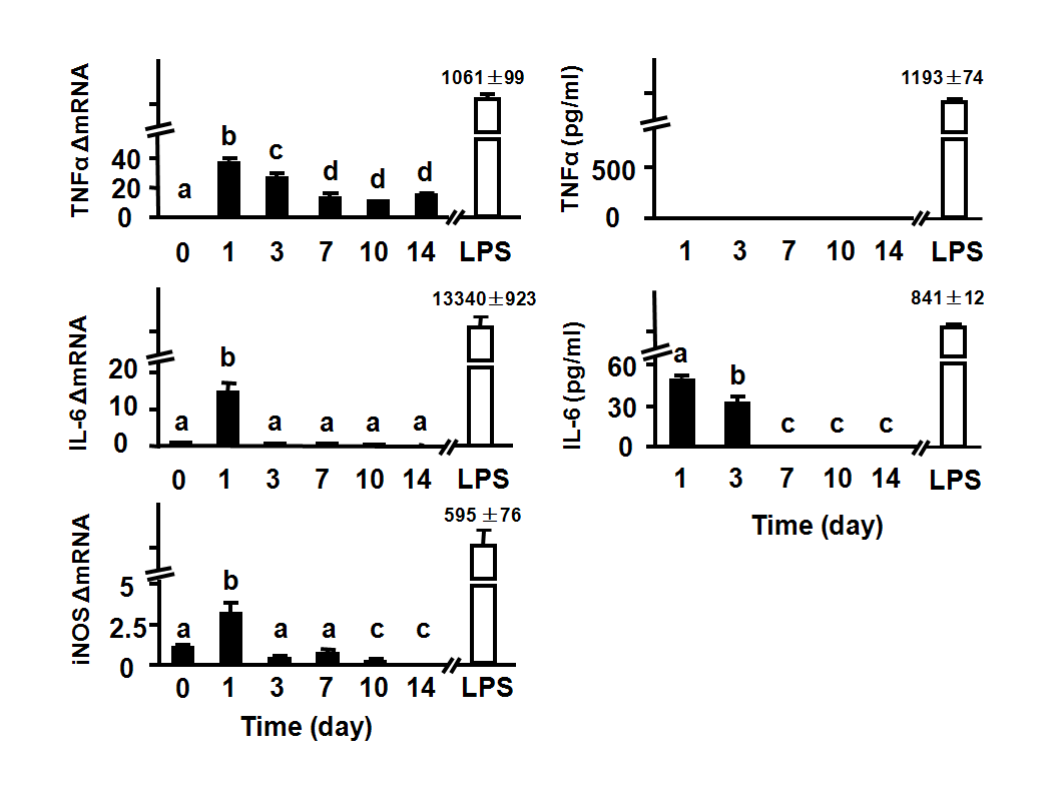
**Proinflammatory cytokines and iNOS expression in hippocampal slices at the mRNA and protein level**. Day 0 represents freshly-isolated hippocampi that were subjected to transversal slicing and mRNA being prepared immediately following slicing. Culture media and slice tissue were collected on days 1, 3, 7, 10 and 14 after the start of the culture. LPS (10 ng/ml) was used as a positive control on day 10, with mRNA and protein being measured 6 h later. Steady-state expression of mRNA transcripts was measured by real-time RT-PCR, and proinflammatory cytokines in the media were measured by ELISA. Average Ct values for at 1, 3, 7, 10 and 14 days for each mRNA species were, respectively: TNFα: 27.6 ± 0.7, 27.8 ± 0.5, 29.0 ± 0.3, 29.1 ± 0.5, 28.7 ± 0.6; IL-6: 30.7 ± 0.4, 34.8 ± 0.2, 36.1 ± 0.9, 35.8 ± 0.8, 36.6 ± 1.1; iNOS: 28.8 ± 0.8, 31.8 ± 0.8, 31.1 ± 1.0, 32.9 ± 0.3, 35.5 ± 0.8. Data represent the mean ± SEM (n = 3 in each group). Bars labeled with different letters (a, b, c or d) are significantly different from each other at *p *< 0.05.

Immediately after transversal slicing on day 0, mRNA from hippocampi was isolated. Although mRNA for TNFα, IL-6 and iNOS could be detected immediately after slicing on day 0, the amount was extremely low, particularly compared to the amount induced by LPS later in culture (Fig. 3). Indeed, after 10 days in culture, treatment with LPS dramatically increased expression of TNFα, IL-6 and iNOS mRNA and induced roughly a nanogram per milliliter secretion of TNFα and IL-6 protein in the culture medium (Fig. 3). The amount of mRNA and protein caused by transversal slicing was minimal compared to treatment with LPS, but nonetheless, was detectable in several instances. For example, TNFα mRNA increased on both days 1 and 3 (*p *< 0.01) after slicing, but it returned to low but detectable levels by day 7. However, the increase in amplified mRNA for TNFα was not accompanied by any detectable TNFα released into the medium at any time point. There was a temporary increase in IL-6 mRNA on day 1 (Fig. 3, *p *< 0.01). IL-6 protein in the culture medium was significantly increased on days 1 and 3 (*p *< 0.01). Expression of iNOS mRNA was only slightly increased (3-fold; *p *< 0.01) on day 1. This modest increase in iNOS mRNA was not associated with any increase in nitrite levels in the culture medium, as measured by Griess reaction (data not shown).

**Figure 4:**
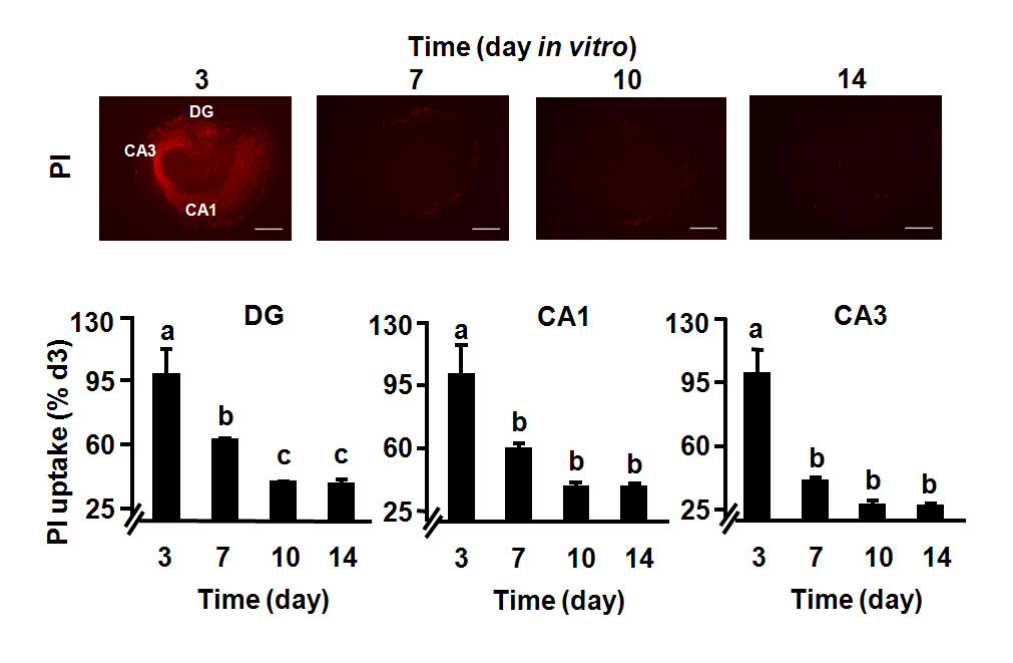
**Time course of cell death in OHSCs**. The OHSCs were treated with PI at different time points after the start of culture. Red fluorescence indicates the dead-cell population using PI staining. Bars represent the mean ± SEM (n = 3 in each group). Bars labeled with different letters (a, b or c) are significantly different from each other at *p *< 0.05. Scale bar = 500 μm.

The viability of OHSCs was determined by PI staining [44]. As shown in Fig. 4, dead/dying cells could be detected in the dentate gyrus (DG), CA1 and CA3 regions (p < 0.01) during the first 3 days of OHSCs culture. This was followed by a gradual decline in the proportion of PI-positive cells. Quantitative analysis confirmed that PI uptake significantly increased on day 3 (*p *< 0.01) and then remained at rather low levels from days 7 through 14. The OHSCs responded very well to LPS at day 10 (Fig. 3), so the decline in proinflammatory cytokine expression that occurred with increasing time in culture was very unlikely to have been caused by cell death. Collectively, results of these experiments confirm that transversal slicing of the hippocampus causes an acute inflammatory response. These data also establish that OHSCs almost entirely recover from the explantation procedure within 7 days.

### Organotypic hippocampal slice cultures respond to LPS by increased expression of proinflammatory cytokines

**Figure 5:**
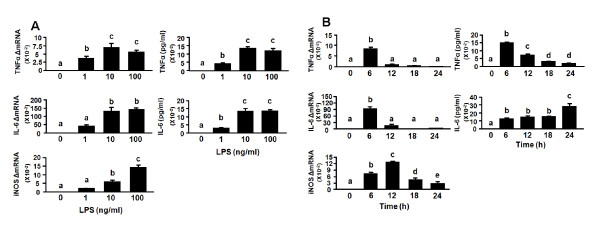
**LPS induces proinflammatory cytokines and iNOS expression in OHSCs in a dose- and time-dependent manner**. (A) 1, 10 or 100 ng/ml LPS were added to the medium after 10 days in culture. Tissue and media were collected 6 h later. Average Ct values for 1, 10 or 100 ng/ml LPS were, respectively, for TNFα: 20.6 ± 0.6, 19.6 ± 0.5, 19.6 ± 0.6; IL-6: 24.1 ± 0.6, 22.5 ± 0.6, 22.1 ± 0.6; iNOS: 26.2 ± 0.9, 24.9 ± 1.0, 23.6 ± 0.9. (B) Hippocampal slices were treated with LPS (10 ng/ml) and tissue and media were collected at various times. Average Ct values at 6, 12, 18 and 24 h were, respectively, for TNFα: 19.7 ± 0.1, 22.8 ± 0.7, 24.4 ± 0.9, 25.3 ± 0.7; IL-6: 23.2 ± 0.3, 24.9 ± 0.7, 28.1 ± 1.1, 28.8 ± 0.7; iNOS: 24.1 ± 0.1, 22.7 ± 0.6, 24.4 ± 1.1, 25.2 ± 1.0. Amount of the proinflammatory cytokines was measured by ELISA. Bars represent the mean ± SEM (n = 3 in each group). Bars labeled with different letters (a, b, c, d or e) are significantly different from each other at *p *< 0.05.

Based on the *in vivo *response to central injection of LPS (Fig. 2), the time point of 6 h was selected for carrying out dose-response experiments to determine the effect of LPS on cytokine expression. Slices were exposed to 1, 10 and 100 ng/ml of LPS on day 10 in culture. As shown in Fig. 5A, LPS increased TNFα and IL-6 production at both the mRNA (*p *< 0.05) and protein (*p *< 0.01) levels in a dose-dependent manner, with a maximum at 10 ng/ml. In control cultures containing medium alone, neither TNFα nor IL-6 could be detected. iNOS mRNA reached a maximum at 100 ng/ml LPS (*p *< 0.01) but this was not associated with a detectable increase in nitrite levels at the 6 h time point (data not shown). Nitrite levels remain undetectable in control OHSCs through at least 13 days in culture. Following LPS stimulation, nitrites begin to rise to detectable levels only after 48 h, which allows adequate amounts to accumulate in the medium (data not shown).

For kinetic studies, OHSCs were exposed to 10 ng/ml LPS for 6, 12, 18 and 24 h on day 10 of culture. As shown in Fig. 5B, the greatest expression of both mRNA and protein for TNFα occurred at 6 h (*p *< 0.01). In contrast, IL-6 mRNA peaked at 6 h (*p *< 0.01) and gradually returned to the control level at 24 h. IL-6 concentration in the culture medium increased after 6 h (*p *< 0.01) and reached a maximum at 24 h (*p *< 0.01). iNOS mRNA increased at 6 h (*p *< 0.01) and peaked at 12 h (*p *< 0.01), but there was no concomitant increase in nitrite levels at any time point. These results reflect the capability of OHSCs to respond to inflammatory stimuli in a manner similar to that which occurs *in vivo*, indicating that OHSCs represent a reliable and valuable model for investigating neuroimmune interactions in a mouse system.

### LPS induces expression of IDO in the absence of IFNγ transcripts in murine OHSCs

**Figure 6:**
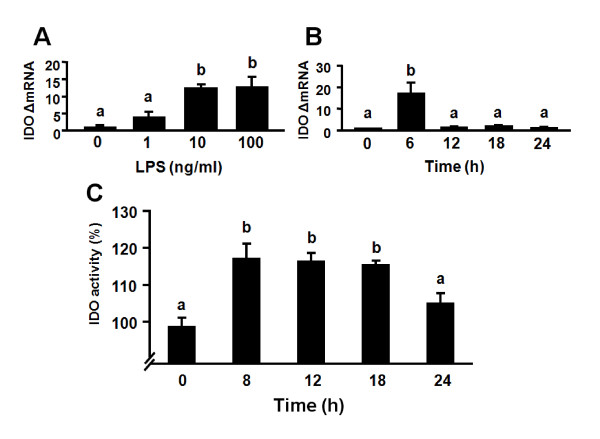
**LPS increases IDO mRNA and enzymatic activity in OHSCs**. (A) 1, 10 or 100 ng/ml LPS were added to the medium after 10 days in culture. Tissue and media were collected 6 h following addition of LPS. Average Ct values for 1, 10 or 100 ng/ml LPS for IDO were, respectively, 38.4 ± 0.8, 36.4 ± 0.3, 36.4 ± 0.3. (B) Hippocampal slices were treated with LPS (10 ng/ml) for various times. Average Ct values at 6, 12, 18 and 24 h for IDO were, respectively, 36.7 ± 0.4, 39.5 ± 0.5, 39.7 ± 0.4, 40 ± 0. (C) Hippocampal slices were treated with LPS (10 ng/ml) for 8, 12, 18 and 24 h. IDO enzymatic activity was determined by measuring the amount of kynurenine formed upon incubation of slices lysates with exogenous L-tryptophan. IDO enzymatic activity was expressed as a percentage of the medium control. Bars labeled with different letters (a or b) are significantly different from each other at *p *< 0.05.

We recently established that LPS induces expression of IDO in primary murine microglial cells in the absence of any detectable IFNγ [37]. Here we used real-time RT-PCR to determine whether LPS induces IDO steady-state transcripts in OHSCs as it does *in vivo *at 6 h. To determine an optimal dose for LPS-induced expression of IDO, OHSCs were treated with 1, 10 and 100 ng/ml of LPS for 6 h. As shown in Fig. 6A, IDO mRNA expression could be detected at 1 ng/ml LPS and reached a maximum at 10 ng/ml LPS (*p *< 0.05). Kinetic studies were then carried out in which OHSCs were exposed to LPS (10 ng/ml) for 6, 12, 18 and 24 h. Figure 6B shows that IDO mRNA could not be detected in OHSCs prior to addition of LPS (40 amplification cycles). However, IDO expression was strongly but transiently induced by LPS at 6 h (*p *< 0.01), returning to the same level as time 0 by 12 h. Importantly, this LPS-induced expression of IDO did not require endogenous synthesis of IFNγ because no IFNγ mRNA could be detected at any time point in LPS-stimulated slices (40 amplification cycles, data not shown). Similarly, we found that LPS did not cause a statistically significant increase in IFNγ mRNA expression at the earlier time point of 2 h (data not shown). This finding using OHSCs is consistent with the results of recent studies reporting that IFNγ-independent mechanisms can mediate IDO induction in hippocampal neurons [45] and primary murine microglial cells [37].

### LPS increases IDO enzymatic activity in OHSCs

To assess whether OHSCs express functional IDO, we determined IDO enzymatic activity by measuring the amount of kynurenine formed following incubation of OHSCs lysates with exogenous L-tryptophan (Fig. 6C). These experiments showed that IDO enzymatic activity increased with time following exposure to LPS. IDO enzymatic activity was augmented by LPS at 8 h (*p *< 0.01), and this functional activity remained elevated at 12 and 18 h (*p *< 0.01) of culture compared to medium control. By 24 h, IDO enzymatic activity returned to baseline. These findings are consistent with our previous data showing that LPS-induced increases in brain IDO mRNA precede activation of IDO and are associated with increased IDO enzymatic activity *in vivo *[14] and in primary murine microglia [37].

## Discussion

Experiments in this report were designed to answer three major questions: (1) Can a direct central challenge with LPS activate IDO in the hippocampus and induce depressive-like behavior? (2) Can a murine organotypic culture system serve as a reliable *in situ *model for investigating mechanistic aspects of the depressive effects of inflammation? (3) Does this *in situ *model of OHSCs respond like primary microglia *in vitro *where IDO induction can occur via an IFNγ-independent mechanism? To our knowledge, this is the first report to demonstrate that centrally administered LPS induces depressive-like behavior as measured by increased immobility in the forced swim test after sickness behavior has dissipated and this effect is associated with IDO activation. Furthermore, the effect of LPS on IDO production *in vivo *translates to the *in situ *setting, and IDO activation in response to LPS occurs in the absence of up-regulation of transcripts for IFNγ. Thus, murine OHSCs can be reliably used to study the mechanisms of cytokine-induced activation of IDO without interference of cytokine synthesis due to preparation of the slices.

Activation of brain cytokine signaling in response to peripheral or central administration of LPS has repeatedly been demonstrated to mediate LPS-induced sickness behavior [2,6,46]. In the present study, *i.c.v*. LPS induced sickness behavior, as measured by body weight loss and decreased social exploration, and this effect was associated with increased expression of steady-state transcripts for TNFα, IL-6, iNOS and IDO in the hippocampus. Moreover, *i.c.v*. administration of LPS was sufficient to induce depressive-like behavior, as measured by increased immobility in the forced swim test when social exploration had dissipated. These results are an important conceptual advance since the association between LPS-induced depressive-like behavior and activation of hippocampal IDO indicates that kynurenine does not need to enter the brain from the periphery to induce depressive-like behavior.

The choice of the hippocampus as a target structure for *ex vivo *analysis of cytokine expression in response to LPS was not motivated by consideration of the possible role of this brain area in major depressive disorders [27,47,48] but by the need to compare *ex vivo *data to *in situ *data collected in OHSCs. Compared to primary cultures of brain cells, OHSCs have the advantage of conserving some form of neuroanatomical organization together with functional cell-to-cell interactions [32,33]. OHSCs recreate an intact brain microenvironment that preserves the cytoarchitecture and functional information-processing properties of neurons. The morphological characteristics of neurons and glial cells in hippocampal slice cultures have been studied by both light and electron microscopy. During slice preparation, cutting of afferent and efferent axons inevitably leads to a rearrangement of connectivity over the following days. Most cells on the slice surface are healthy, receiving and sending inputs from intact axons, and both increased excitatory miniature synaptic current frequency and dendritic complexity are established within the first week of culture [49]. During the first 2 days of culture, astrocytes appear thin and elongated in the rat system. Over the next 8 days, astrocytes become hypertrophic and form a gliotic scar over the surface of the culture. "Reactive/activated" microglia reaches peak numbers immediately after injury induced by culture preparation. These ameboid microglia are replaced after 10 days in culture by "resting/ramified" microglia [50]. Since long term organotypic cultures of the murine hippocampi have been rarely used in neuroimmunology, it was necessary first to validate this model system. The early but transient increases in TNFα and IL-6 expression and release in response to the traumatic injury caused by transversal sectioning probably originated from activated microglial cells. After several days *in vitro*, the decline in the production of cytokines to baseline almost certainly reflected the return of microglia to their non-activated phenotype. However, this conclusion still needs to be confirmed using appropriate techniques.

Most dead cells disappeared and cytokines could no longer be detected in the culture medium after the first week of culture. This finding indicates that the inflammatory profile has certainly subsided, such that murine OHSCs can be safely used as soon as after 7 days in culture. In the rat system, the optimal time window for experiments is between day 6 and day 18 [51]. At this time, most of the slices are healthy and vital. After this time, the percentage of usable slices decreases, but viable rat OHSCs can last a minimum of 6-8 weeks in culture [51]. In the present conditions of murine OHSCs, LPS evoked a substantial expression of mRNA as well as the synthesis and release of TNFα and IL-6. These data confirm results previously reported in rat OHSCs [52]. It is important to note that the concentration of LPS-induced TNFα and IL-6 production were significantly higher than the levels measured at the onset of the organotypic culture.

Glial cells are the main sources of cytokines in CNS injury and inflammation [53]. TNFα produced at the earlier time point of 6 h probably originates from activated microglia whereas the peak in IL-6 that occurred at 24 h is produced by astrocytes that are subsequently activated by TNFα [53]. We did not report changes in IL-1β mRNA or protein in OHSCs since the release of IL-1β in the culture medium requires the addition of ATP (data not shown). This implies that the production of IL-6 was likely solely dependent on TNFα production.

We were most interested in the potential use of murine OHSCs to determine whether LPS can activate IDO in this *in situ *system. We previously demonstrated that IDO, the first and rate-limiting enzyme in the synthesis of kynurenine from the precursor of tryptophan, is a required mediator of depressive-like behavior in response to LPS in mice [14]. The increase in brain IDO activity is invariably preceded by enhanced expression of IDO mRNA, which can therefore be used as a surrogate marker of IDO activation [14,37]. Expression and activation of IDO in response to LPS and other stimuli are usually studied in various types of transformed and primary cell cultures. However, murine OHSCs cultures have never been used for this purpose despite their potential advantages for the study of the intricate cell-to-cell communication mechanisms that mediate activation of IDO in the brain. *In situ *hybridization studies of the expression of IDO in the brain in response to LPS indicate that endothelial cells are the likely source of brain IDO [14] whereas immunohistochemistry findings point to a possible neuronal localization [54]. IDO is expressed in all types of brain cells that have been studied so far, including endothelial cells, glia and neurons [18]. The fate of IDO-produced kynurenine is not the same in microglia and other cell types because of differences in the intracellular enzymes that metabolize kynurenine [20]. Therefore, it is obviously important to determine in future experiments which brain cell types are predominantly involved in the production and degradation of kynurenine in response to an immune stimulus.

IFNγ is considered to be the prototypical inducer of IDO in a variety of cell types [55] as well as in clinical situations in which inflammation-associated depression occurs [11]. However, IFNγ-independent activation pathways have also been reported in response to LPS [56,57]. LPS-induced upregulation of IDO in the brain parallels increased IFNγ mRNA expression *in vivo *[14] but whether this leads to increased mature IFNγ protein can be disputed [58]. This does not mean that brain macrophages and microglia are capable of producing IFNγ in other circumstances, even if most IFNγ is normally produced by T lymphocytes and NK cells [59].

In conclusion, results of the present study clearly demonstrate that direct, central activation of brain cytokine signaling and IDO is associated with development of depressive-like behavior in the mouse. These results provide further arguments for the targeting of brain IDO to alleviate inflammation-associated depression.

## Competing interests

RD has received honorarium from Astra-Zeneca, Bristol-Myers-Squibb and Lundbeck Laboratories. He is working as a consultant for Lundbeck Laboratories. KWK has received honorarium from Astra-Zeneca.

## Authors' contributions

XF and SMZ developed the technical procedures for OHSCs, assisted by advice on treatment application from JCO and AK. JCO was responsible for analyzing the *in vivo *component of these experiments. XF designed the experiments with the help of RD and KWK, performed the OHSCs experiments, analyzed the results and drafted the manuscript. RD and KWK secured funding for the project and helped with the final version of the manuscript. All authors read and approved the final manuscript.
